# Sustainable valorization of recycled cellulose fibers into boric acid/silica modified cementitious composites *via* multi-response optimization

**DOI:** 10.1039/d6ra04542a

**Published:** 2026-07-09

**Authors:** Fehmi Saltan, Mücahit Uğur, Ayça Günay, Medine Nur Karakurt, Fatih Turan, Mehmet Özdoğan, Şahin Güleryüz

**Affiliations:** a Faculty of Science, Department of Chemistry, Cankiri Karatekin University Çankırı Turkey fehmisaltan@karatekin.edu.tr; b Department of Chemistry Engineering, Faculty of Engineering, Cankiri Karatekin University Çankırı Turkey; c Department of Mining Engineering, Faculty of Engineering, Dokuz Eylul University İzmir Türkiye

## Abstract

Recycled cellulose fibers recovered from paper industry waste were valorized as sustainable reinforcement materials for the development of boric acid/silica-modified cementitious composites with balanced mechanical and thermal performance. A Taguchi–TOPSIS multi-response optimization approach was employed to systematically evaluate the combined effects of cellulose fiber, boric acid, and silica contents on compressive strength, heat capacity, thermal conductivity, and water absorption. Structural characterization by FT-IR, XRD, and SEM-EDX confirmed that the principal cement hydration phases were preserved after modification, while silica-rich formulations exhibited a relatively denser and more homogeneous matrix morphology. Thermogravimetric analysis showed that cellulose incorporation increased the total mass loss from approximately 12–14% for the reference cement to 17–20% for the modified composites, whereas boric acid and silica partially moderated this effect by maintaining residual mass values of 80–85% at elevated temperatures. Among the experimentally evaluated formulations, the Taguchi–TOPSIS multi-response optimization identified the A2B2C3 composition as the optimum formulation, achieving a compressive strength of 38.73 MPa, corresponding to a 52.63% improvement over the reference mixture, together with a 37.06% increase in heat capacity, while simultaneously reducing thermal conductivity and water absorption. These results demonstrate that the combined incorporation of recycled cellulose fibers, boric acid, and silica provides an effective strategy for developing sustainable cementitious composites with improved multifunctional performance while promoting the high-value utilization of paper industry waste.

## Abstract 2

Taguchi–TOPSIS optimization enabled the development of recycled cellulose fiber/boric acid/silica cementitious composites with superior mechanical, thermal, and microstructural performance for sustainable construction applications.

## Graphical abstract

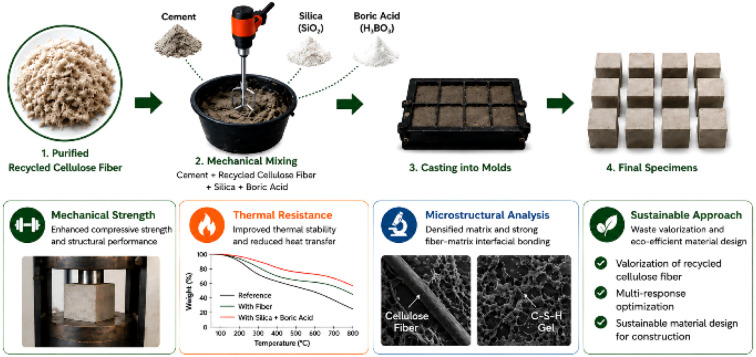

## 1. Introduction

The rapid growth of urbanization and infrastructure development has led to a continuous increase in the consumption of cement-based construction materials. Despite their widespread use owing to durability and mechanical reliability, conventional cement production remains one of the major industrial sources of energy consumption and CO_2_ emissions.^1–3^ Accordingly, current sustainable material strategies increasingly focus not only on alternative binders, but also on the integration of recycled or waste-derived components into existing cementitious systems in order to improve resource efficiency and reduce environmental burden.^4,5^ Within this context, cellulose fibers recovered from paper recycling processes represent an abundant yet underutilized secondary resource. Although generally treated as industrial waste, these lignocellulosic fibers offer a promising reinforcement phase for cement-based matrices while simultaneously supporting circular economy principles and waste reutilization pathways in construction materials.^6–9^

Cellulose fibers are known to improve crack-bridging ability, toughness, and micro-reinforcement efficiency in cementitious composites.^10–12^ Nevertheless, the beneficial contribution of recycled cellulose strongly depends on fiber dispersion, surface cleanliness, and interfacial compatibility with the surrounding inorganic matrix. Inadequate dispersion or weak fiber–matrix adhesion may restrict stress transfer, generate localized heterogeneity, and ultimately limit the expected reinforcing efficiency.^13–16^ Therefore, the sustainable utilization of recycled cellulose fibers requires not only their physical incorporation into cement matrices, but also an accompanying matrix engineering strategy capable of stabilizing the resulting organic–inorganic composite structure.

To address these limitations, mineral and functional additives are frequently introduced to improve the integrity and performance stability of cementitious systems.^17–19^ Among them, silica-based additives are particularly important because they contribute to particle packing, promote matrix densification, support C–S–H development, and reduce pore-related discontinuities within the hydrated cement network.^20,21^ In parallel, boron-based additives such as boric acid have attracted attention as thermally moderating and structurally regulating components in composite materials due to their contribution to thermal resistance and decomposition control.^22,23^ Although these functionalities have been separately discussed in different composite systems, their combined use together with recycled cellulose fibers in a unified cementitious platform remains insufficiently understood from a sustainable material design perspective.

As summarized in Table 1, previous sustainability-oriented studies have extensively investigated cellulose-based fiber reinforcement, silica-containing mineral additives, and boron-based modifiers in cementitious materials. However, comparatively fewer studies have systematically evaluated their combined influence using an integrated Taguchi–TOPSIS multi-response optimization framework to simultaneously optimize multiple performance characteristics. In particular, the combined influence of recycled cellulose fibers, silica, and boron-based additives on the mechanical, thermal, and microstructural performance of cementitious composites has not yet been systematically evaluated using a multi-response optimization strategy. This lack of an integrated assessment limits the rational design of waste-derived cementitious materials with balanced multifunctional properties. Therefore, the present study aims to sustainably valorize recycled cellulose fibers recovered from paper industry waste into boric acid/silica-modified cementitious composites while systematically evaluating the combined effects of cellulose fibers, boric acid, and silica through a Taguchi–TOPSIS multi-response optimization approach. Compared with previous studies, which have predominantly examined the individual effects of fibers or mineral additives, the present work provides a comprehensive evaluation of their combined contribution to the development of resource-efficient cementitious composites with improved mechanical, thermal, and durability-related performance. The findings contribute to a better understanding of integrated fiber–additive systems and provide a practical waste-to-value strategy for the design of eco-efficient cementitious materials.

**Table 1:** Literature-guided sustainability strategies and material design gaps for recycled fiber modified cementitious composites

Theme	Brief description	Relevance to the present study	References
Net-zero targets in the cement sector	Reduction of clinker demand, alternative fuels, carbon capture technologies, and low-emission material strategies are widely recognized routes for decreasing the environmental footprint of cement production	Establishes the broader decarbonization motivation for developing waste-integrated cementitious materials	1–4
Eco-efficient cements	Material-efficient formulations and partial replacement approaches provide practical pathways to improve sustainability without completely replacing Portland cement	Supports additive-assisted sustainable redesign of conventional cementitious systems	2 and 5
SCMs and alternative binders	Slag, calcined clay, limestone, and alkali-activated systems are extensively explored as environmentally favorable binder alternatives	Positions the present work within broader sustainable cement material development while retaining a conventional binder matrix	1–3 and 6
Value-chain improvements	Optimized material design, production efficiency, and performance-oriented formulations can significantly reduce the life-cycle burden of cement-based products	Highlights the importance of compositional optimization for resource-efficient cementitious materials	2–5
Fiber reinforcement in cementitious systems	Natural and synthetic fibers, particularly cellulose-based fibers, are used to improve crack resistance, toughness, and microstructural continuity	Provides the basis for valorizing recycled cellulose fibers as a reinforcing secondary resource	7–10
Mineral/additive modification of cement systems	Silica, boron compounds, and related additives are employed to enhance matrix densification, thermal response, and overall structural stability	Supports the functional role of boric acid and silica in regulating the composite matrix	18–23
Contribution relative to previous studies	Although numerous studies have separately investigated cellulose fibers, silica, or boron-containing additives, comparatively fewer studies have systematically optimized their combined influence using an integrated multi-response optimization framework	Defines the unresolved literature gap addressed by the present waste-derived organic–inorganic composite design	1–23

## 2. Materials and methods

### 2.1. Materials

Waste cellulose fiber was supplied by Levent Paper Industry and Trade Inc. (Izmir, Türkiye). A pozzolanic cement, CEM IV/B (P) 32.5 R, was used as the primary binder material for the preparation of the cementitious composites. The cement possesses a Blaine fineness of 5100 cm^2^ g^−1^, a specific gravity of 2.75 g cm^−3^, and a bulk density of 870 g L^−1^. The measured volume expansion was 1 cm, while the Portland clinker content was approximately 59.0%. The initial and final setting times were determined as 190 and 230 min, respectively. The compressive strengths of the neat cement at curing ages of 2 and 28 days were 14 and 37 MPa, respectively.

To improve the workability of the mixtures, a polycarboxylate ether-based superplasticizer (BASF Master Glenium 608) was used. The admixture was supplied in liquid form with a light brown appearance and a specific gravity ranging from 1.059 to 1.099 kg L^−1^. The chloride and alkali contents of the product were below 0.1% and 3%, respectively. Silicon dioxide (SiO_2_, acid washed) and boric acid (H_3_BO_3_, Technipur grade, powder) were purchased from Sigma-Aldrich and used as received without further purification.

### 2.2. Instrumentation

Thermogravimetric (TG) analyses were carried out using a PerkinElmer Diamond TA/TGA instrument in the temperature range of 25–600 °C at a heating rate of 10 °C min^−1^ under a nitrogen flow of 100 mL min^−1^. The sample masses were maintained between 6 and 10 mg. Fourier transform infrared (FT-IR) spectra were recorded on a PerkinElmer Spectrum One-B spectrometer. X-ray diffraction (XRD) analyses were performed using Cu-Kα radiation (*λ* = 1.5406 Å) over an appropriate 2*θ* scanning range at a scan speed of 0.1° s^−1^. Scanning electron microscopy (SEM) images were obtained using a field-emission scanning electron microscope (Thermo Scientific Apreo S SEM) operated under high vacuum at an accelerating voltage of 15 kV and a working distance of 6.0 mm. Prior to SEM and EDX analyses, the samples were sputter-coated with an Au/Pd alloy layer to improve surface conductivity. Elemental compositions were determined by energy-dispersive X-ray (EDX) spectroscopy under the same operating conditions. Compressive strength measurements were performed using a universal compression testing machine (ELE International, Autotest 3000) with a maximum loading capacity of 3000 kN. The load was applied at a controlled rate of 0.5–1.0 MPa s^−1^, and the compressive strength values were calculated from the ratio of the measured failure load to the specimen cross-sectional area. Thermal conductivity and specific heat capacity measurements were conducted using a Decagon KD2 thermal analyzer based on the transient hot-wire method at room temperature in accordance with ASTM D5334. Thermal conductivity was measured using a TR-1 single probe (10 cm), whereas specific heat capacity was determined using SH-1 dual probes (3 cm) inserted into pre-drilled holes in the specimens.

### 2.3. Waste fiber pretreatment and preparation

**Fig. 1:**
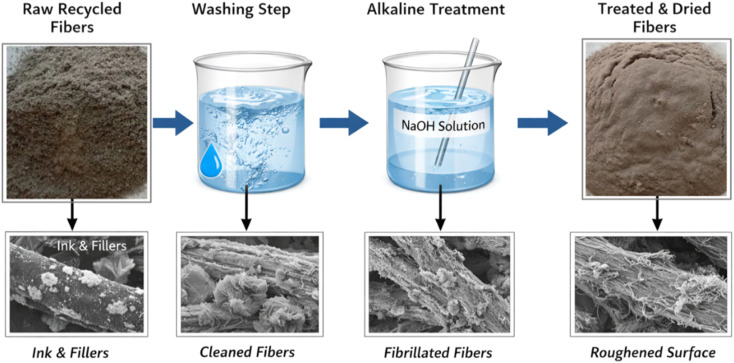
Pretreatment sequence of recycled cellulose fibers showing washing, alkaline activation, drying, and representative SEM surface morphology changes.

Prior to incorporation into the composite mixtures, the recycled cellulose fibers were subjected to a pretreatment process to improve surface cleanliness and dispersion behavior, as schematically illustrated in Fig. 1. Initially, the fibers were thoroughly washed with water to remove loosely attached contaminants, including ink residues, fine mineral particles, and coating remnants originating from the paper recycling process. Subsequently, the fibers were subjected to an alkaline activation treatment using a 2 wt% NaOH solution at room temperature for 1 h to partially remove surface impurities and enhance the accessibility of hydroxyl (–OH) groups on the cellulose surface. After the alkaline treatment, the fibers were thoroughly rinsed several times with distilled water until a neutral pH was reached to ensure the complete removal of residual alkali, followed by drying at 70 °C prior to use. The dried fibers were then passed through a 200-mesh sieve (75 µm). This sieve size was selected because it represented the predominant fiber fraction obtained after the pretreatment process while providing a relatively uniform particle size distribution that facilitated homogeneous dispersion of the fibers within the cementitious matrix. Finally, the sieved fibers were stored in an airtight container until further processing. Additional photographs of the pretreatment stages are presented in SI Fig. S1.

### 2.4. Preparation of cellulose fiber–reinforced cement composites

A Taguchi experimental design methodology was employed to prepare and evaluate the recycled cellulose fiber reinforced cementitious composites investigated in this study. The reference mixture consisted of 1400 g cement, 490 g water, and a polycarboxylate-based superplasticizer. In the mixing procedure, the superplasticizer was first dispersed homogeneously in a portion of the mixing water and then introduced into the cement. Recycled cellulose fibers were subsequently added and mixed thoroughly to ensure uniform distribution within the matrix. Finally, boric acid and silica were incorporated into the main mixture, and mixing was continued until a homogeneous paste consistency was achieved.

**Fig. 2:**
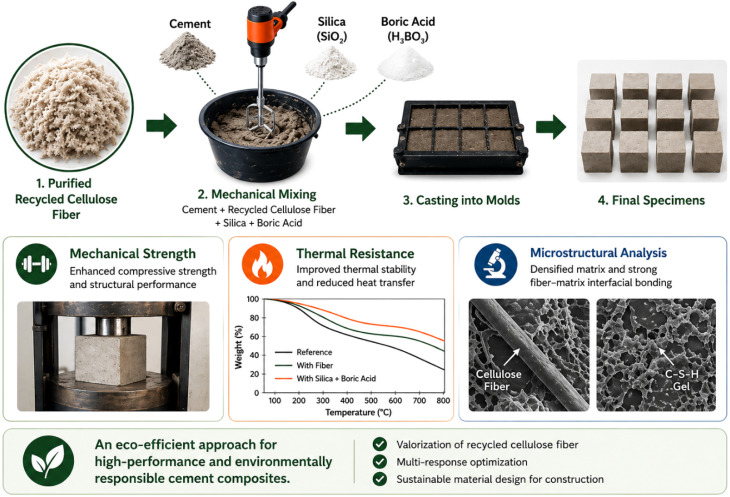
Integrated schematic illustration of composite preparation, molding, curing, and representative performance characteristics of recycled cellulose fiber reinforced cementitious composites.

An L9 orthogonal array was adopted to examine the effects of three key formulation parameters on composite performance. The resulting mixtures were cast into steel cube molds (5 × 5 × 5 cm) for compressive strength, specific heat capacity, thermal conductivity, and water absorption measurements, while silicone molds (2 × 2 × 2 cm) were used for microstructural characterization. After casting, the specimens were kept in the molds for 24 h, demolded, and conditioned at room temperature before being subjected to a 28 day curing period. At the end of curing, the samples were used for all subsequent experimental analyses. To ensure repeatability, each of the nine experimental formulations defined by the Taguchi design was prepared and tested in duplicate. The principal stages of the composite preparation and curing procedure are illustrated in Fig. 2.

### 2.5. Multi-response optimization methodology

A Taguchi-based experimental design coupled with the TOPSIS decision-making approach was employed to optimize the multifunctional performance of the recycled cellulose fiber reinforced cementitious composites. The Taguchi methodology enables the systematic evaluation of multiple formulation variables with a reduced number of experimental runs, thereby minimizing experimental time and material consumption. In the present study, an L9(3^3^) orthogonal array was selected to investigate the effects of three independent parameters at three different levels. The Taguchi signal-to-noise (S/N) ratio was used to evaluate the quality characteristics according to the “larger-the-better” and “smaller-the-better” criteria, as expressed in eqn (1) and (2), respectively.^24^ Since the conventional Taguchi method evaluates each response individually, a TOPSIS-based normalization procedure was additionally applied to integrate all performance outputs into a single representative closeness coefficient. This hybrid optimization route enables the simultaneous assessment of mechanical, thermal, and durability-related responses within a unified decision framework.^25–27^ Detailed mathematical calculations of the TOPSIS procedure are provided in the SI.1
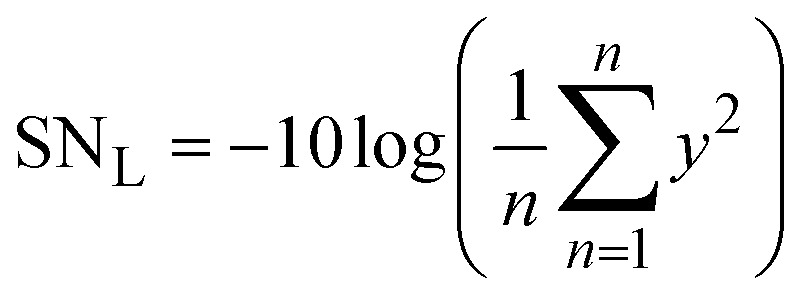
2
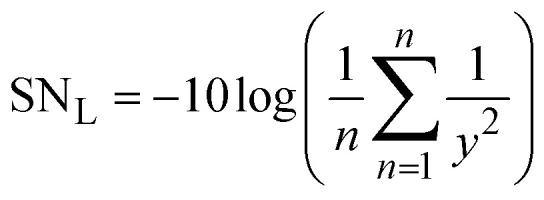


### 2.6. Performance criteria and optimization objectives

To evaluate the overall performance of the developed cementitious composites, four quality criteria were selected: 28 day compressive strength, 28 day specific heat capacity, 28 day thermal conductivity, and 28 day water absorption. Compressive strength was considered the primary indicator of mechanical integrity and was determined according to TS EN 12390-3. Specific heat capacity was selected as an indicator of thermal energy storage capability,^28,29^ whereas thermal conductivity was used to assess the insulation behavior of the composites in accordance with ASTM D5334.^30^ Water absorption, determined according to EN 12390-7, was used as a durability-related parameter reflecting the moisture uptake tendency of the matrix.^31,32^

The optimization objective was defined as maximizing compressive strength and heat capacity while minimizing thermal conductivity and water absorption. Equal weighting factors were assigned to all four quality criteria in order to obtain a balanced multifunctional performance assessment. The selected quality criteria and their corresponding normalized weights are summarized in Table 2.

**Table 2:** Quality criteria, optimization objectives, and normalized weighting factors used in the TOPSIS-based multi-response evaluation

Quality criterion	Symbol	Definition	Optimization objective	Normalized weight
Compressive strength (28 day)	R1CS28	Mechanical strength of composite specimens (MPa)	Larger is better	0.25
Specific heat capacity (28 day)	R2CP28	Thermal energy storage capability (MJ m^−3^ K^−1^)	Larger is better	0.25
Thermal conductivity (28 day)	R3T28	Heat transfer coefficient of composite specimens (W m^−1^ K^−1^)	Smaller is better	0.25
Water absorption (28 day)	R4WA28	Moisture uptake tendency of composite specimens (%)	Smaller is better	0.25

### 2.7. Experimental factors and design levels

Three independent formulation parameters affecting the composite performance were selected as the control factors of the experimental design: cellulose fiber content (*A*), boric acid content (*B*), and silica content (*C*). Each factor was evaluated at three different levels, and the corresponding factor definitions are listed in Table 3.

**Table 3:** Control factors and corresponding levels used in the Taguchi experimental design

Symbol	Parameter	Levels		
		1	2	3
*A*	Cellulose fiber (%)	2.5	5	10
*B*	Boric acid (%)	0.5	1	2
*C*	Silica (%)	1	2	3

Based on these factor levels, the coded and uncoded experimental combinations generated according to the L9(3^3^) orthogonal array are presented in Table 4. The resulting nine formulations constituted the basis of the experimental composite preparation and subsequent performance analyses.

**Table 4:** Coded and uncoded formulation combinations generated according to the L9(3^3^) orthogonal array

Sample	Coded levels			Uncoded levels		
	*A*	*B*	*C*	*A* (cellulose fiber amount, g)	*B* (boric acid amount, g)	*C* (silica amount, g)
1	1	1	1	2.5	0.5	1
2	1	2	2	2.5	1	2
3	1	3	3	2.5	2	3
4	2	1	2	5	0.5	2
5	2	2	3	5	1	3
6	2	3	1	5	2	1
7	3	1	3	10	0.5	3
8	3	2	1	10	1	1
9	3	3	2	10	2	2

## 3. Results and discussions

### 3.1. Interfacial compatibility of recycled cellulose fibers within the cementitious matrix

**Fig. 3:**
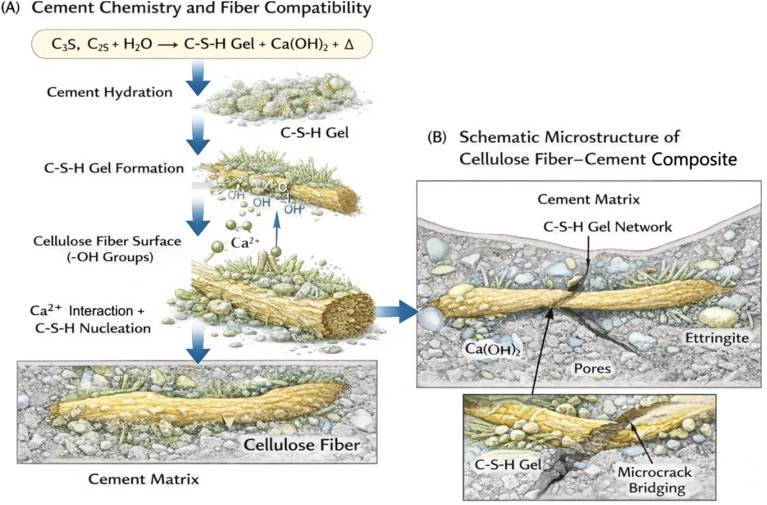
(A) Schematic representation of the proposed interfacial interaction between recycled cellulose fibers and cement hydration products during hydration. (B) Schematic microstructure of the recycled cellulose fiber–cement composite illustrating the C–S–H network, hydration products, pores, and microcrack bridging.

Cellulose is a semi-crystalline natural polymer stabilized by an extensive intermolecular hydrogen-bonding network. The surface of cellulose fibers contains abundant hydroxyl (–OH) groups that play a critical role in fiber–matrix interactions in composite materials. These hydroxyl functionalities provide active sites for hydrogen bonding and weak coordination interactions with inorganic phases, thereby influencing interfacial adhesion and the mechanical integrity of fiber–reinforced systems.^33–35^ The proposed interaction pathway between cellulose fibers and cement hydration products is schematically illustrated in Fig. 3.

Recycled cellulose fibers obtained from paper recycling processes generally contain various surface contaminants originating from pulping, printing, and coating stages. These residues may include printing ink fragments, mineral fillers such as CaCO_3_ and SiO_2_, Al–Si rich deposits, coating remnants, and compact fibrillar agglomerates. The presence of such impurities can partially block accessible hydroxyl groups on the fiber surface and reduce the number of potential bonding sites, thereby weakening interfacial adhesion with the surrounding cementitious matrix and limiting efficient stress transfer.

**Fig. 4:**
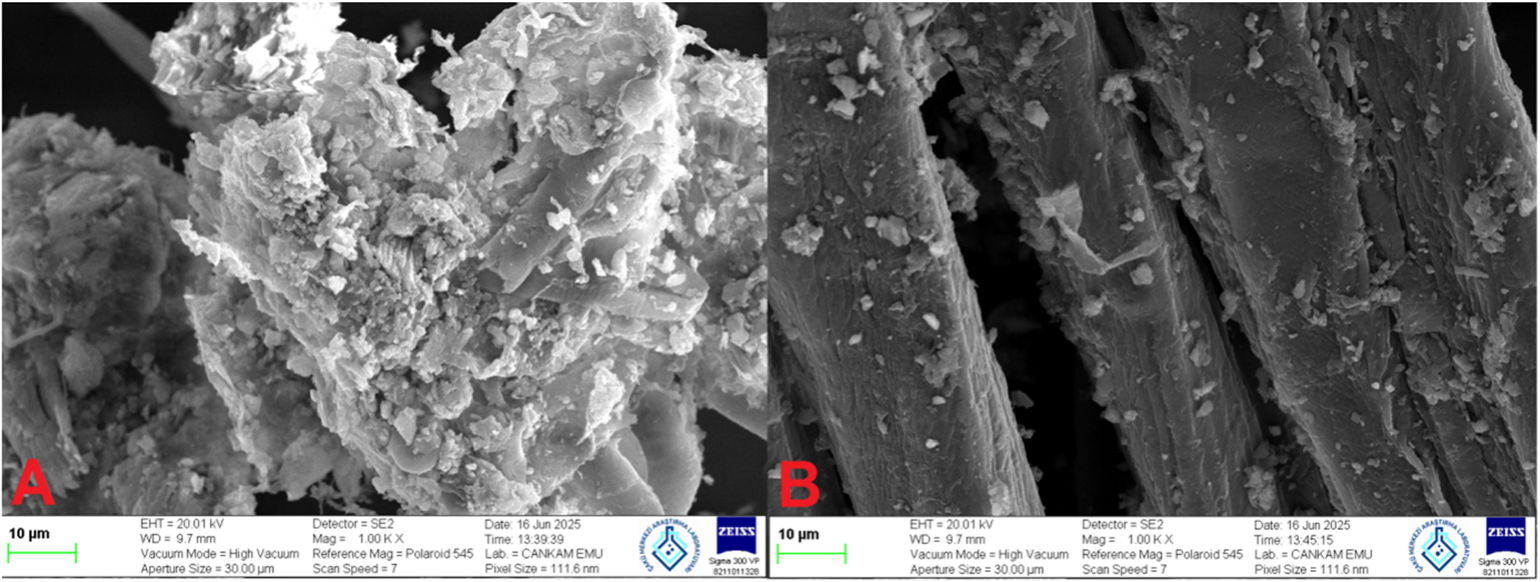
SEM micrographs of recycled cellulose fibers before and after pretreatment: (A) untreated fibers with adhered impurities and mineral residues, and (B) treated fibers with cleaner fibrillar morphology.

For this reason, the recycled cellulose fibers used in this study were subjected to a pretreatment process prior to incorporation into the cement mixtures.^36^ Surface cleaning and mild activation remove loosely attached contaminants while improving the accessibility of hydroxyl-rich regions. In addition, the pretreatment process promotes slight fibrillation, leading to increased surface roughness and a higher potential for mechanical interlocking with hydration products. The morphological differences between untreated and treated fibers are evident in the SEM images presented in Fig. 4. As shown in the micrographs, untreated fibers (Fig. 4A) exhibit adhered mineral particles and surface residues, whereas treated fibers (Fig. 4B) display a cleaner and more exposed fibrillar morphology.^37^ These observations support that the pretreatment process improves the surface availability of cellulose fibers for subsequent matrix interaction.

The effectiveness of the pretreatment process is further supported by the elemental analysis summarized in Table 5. EDX results show that the relative amounts of mineral-associated elements such as Ca, Si, Al, and Mg decrease after cleaning, whereas the carbon content associated with the cellulose backbone increases. For example, calcium decreases from 12.45% to 4.57%, while silicon decreases from 1.47% to 0.40%. Similarly, aluminum and magnesium signals are substantially reduced. These compositional changes indicate the removal of surface-bound inorganic residues and the relative enrichment of cellulose-dominant regions.

**Table 5:** EDX elemental composition of recycled cellulose fibers before and after pretreatment

Metals and minerals found on the surface	Fiber (before processing) %	Fiber (after processing) %
Ca	12.45	4.57
Si	1.47	0.40
Al	1.03	0.13
Mg	0.37	—
C	43.73	60.97
O	40.95	33.85

**Fig. 5:**
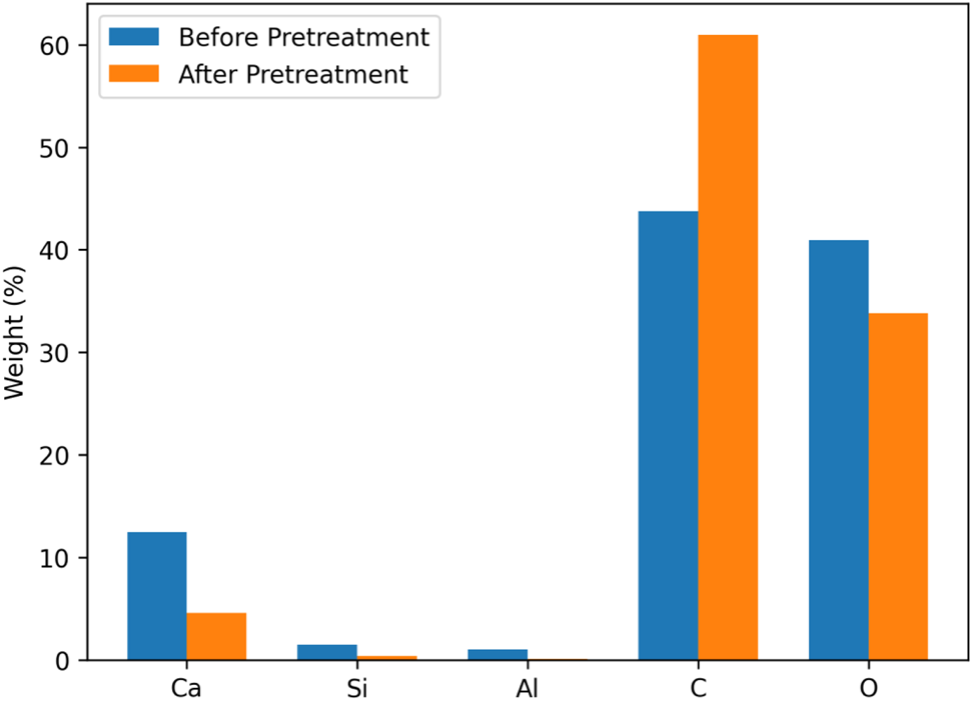
Comparative EDX spectra of recycled cellulose fibers before and after pretreatment, showing the reduction of inorganic mineral residues after surface cleaning.

The compositional differences before and after pretreatment are further visualized by the EDX spectra in Fig. 5, where the reduction of inorganic mineral signals and the relative enrichment of carbon-containing domains can be observed. This surface purification is expected to facilitate a more effective interaction between recycled cellulose fibers and cement hydration products.

The interfacial behavior of recycled cellulose fibers is closely associated with the hydration chemistry of Portland cement, where the hydration of the major silicate clinker phases leads predominantly to the formation of calcium silicate hydrate (C–S–H) gel and calcium hydroxide.^38,39^ Among these products, C–S–H constitutes the principal binding phase of the cementitious matrix and governs the development of the surrounding inorganic microstructure. Within this hydrated environment, cellulose fibers become embedded into an interconnected inorganic network composed of hydration products and capillary domains. The hydroxyl groups present on the fiber surface can interact with calcium-rich regions in the pore solution through weak coordination and hydrogen-bond-assisted adsorption, while the roughened morphology generated by pretreatment provides favorable nucleation sites for local deposition of hydration products.

As hydration progresses, mineral phases tend to accumulate around the fiber surface and create an interfacial transition region that enhances physical anchoring of the fibers within the surrounding matrix. This combined chemical affinity and morphological interlocking improves load transfer across the fiber–matrix interface and supports the micro-reinforcement role of recycled cellulose fibers in the composite structure.^40^

### 3.2. FT-IR analysis

**Fig. 6:**
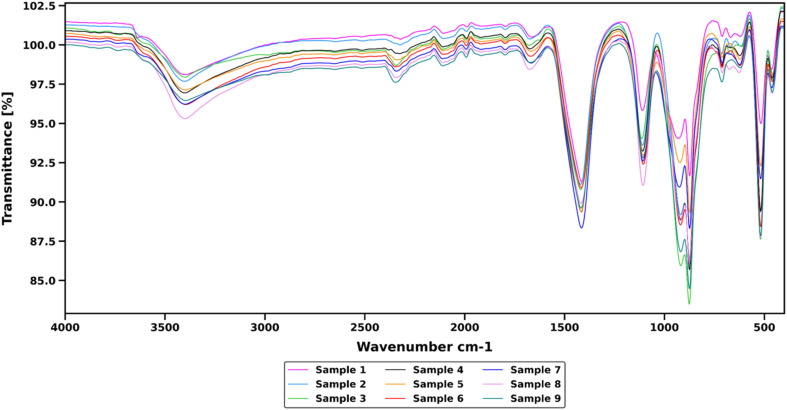
FT-IR spectra of recycled cellulose fiber reinforced cementitious composites modified with boric acid and silica.

The FT-IR spectra presented in Fig. 6 support the coexistence of cement hydration products and cellulose-derived organic functionalities within the modified cementitious composites. A broad absorption band observed in the 3200–3600 cm^−1^ region is attributed to O–H stretching vibrations arising from both bound water in hydration products and hydroxyl groups of cellulose.^41^ The gradual increase in the intensity of this band with higher fiber content is consistent with a greater contribution of hydroxyl-containing domains within the composite structure. The characteristic C–H stretching bands around ∼2900 cm^−1^ further support the incorporation of cellulose fibers into the cement matrix. In addition, the band near ∼1650 cm^−1^ corresponds to H–O–H bending vibrations of adsorbed water, reflecting the hydrated nature of the system.^41^

The carbonate-related bands observed at ∼1400–1450 cm^−1^ indicate partial carbonation,^42^ which is commonly encountered in cementitious materials. More importantly, the strong absorption region between 1000 and 1100 cm^−1^ is assigned to Si–O–Si and Si–O stretching vibrations,^43^ associated with silicate-containing hydration products and the added silica phase. The relatively sharper profile of this region in silica-rich formulations is consistent with a more pronounced silicate contribution within the matrix. The absorption region between 1200 and 1400 cm^−1^ contains overlapping contributions from C–O and Si–O stretching vibrations and may also include B–O-related vibrations reported in the literature.^44^ However, owing to the considerable overlap of these absorption bands and the absence of a control spectrum of boric acid or a boron-only composite, this region cannot be unequivocally assigned to the contribution of boric acid in the present study.

Overall, the FT-IR results suggest that the modified composite structure is primarily governed by the coexistence and interaction of cellulose fibers, silicate-containing phases, and cement hydration products, while the principal cementitious framework remains preserved. Although the spectra support the successful incorporation of the composite constituents, they do not permit direct identification of the individual contribution of each component to the observed spectral features.

### 3.3. SEM-EDX analysis

**Fig. 7:**
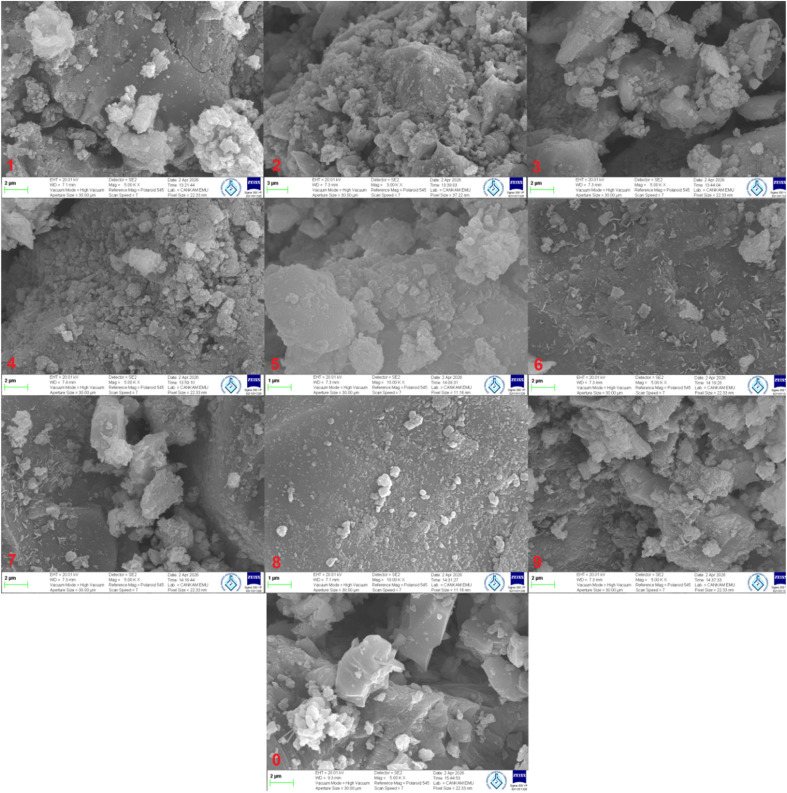
SEM micrographs of the reference cement (sample 0) and recycled cellulose fiber reinforced cementitious composites (samples 1–9) containing different boric acid and silica levels according to the L9 experimental design.

The SEM micrographs presented in Fig. 7 reveal clear morphological differences between the reference cement matrix and the modified composites. The unmodified cement sample (sample 0) exhibits a relatively dense but heterogeneous morphology characterized by irregular hydration domains and localized agglomerated regions, which is typical of plain cement systems. Following the incorporation of recycled cellulose fibers, boric acid, and silica (samples 1–9), progressive microstructural variations are observed depending on the compositional parameters defined in the experimental design. At lower fiber contents (*A* = 1), the matrix remains relatively compact with limited microvoid formation, whereas increasing fiber levels (*A* = 2–3) lead to a more heterogeneous morphology associated with the physical distribution of cellulose-rich interfacial regions within the cementitious matrix. Such heterogeneity may influence local stress transfer and matrix continuity.^45,46^

The influence of silica (*C* factor) is more apparent in samples containing higher silica levels (*C* = 3), where the morphology appears relatively more compact and uniformly distributed. This observation is consistent with improved particle packing and a denser mineral phase distribution within the surrounding matrix, resulting in fewer large discontinuities.^47^ In contrast, samples containing higher boric acid contents (*B* factor) exhibit comparatively more loosely packed and partially agglomerated regions. This morphological variation may be associated with the reported moderating effect of boric acid on cement hydration, which can influence the local development of hydration products and produce a less uniform microstructure.^48^

Overall, the SEM observations suggest that the final morphology of the composites reflects the combined influence of recycled cellulose fibers, silica, and boric acid. The presence of cellulose fibers contributes to localized interfacial heterogeneity within the cement matrix, while silica-rich formulations exhibit a relatively more compact mineral framework. Likewise, the observed morphological variations in boric acid-containing samples are consistent with its reported influence on the hydration process. Although the individual contributions of each component cannot be completely separated based on the present experimental design, the combined observations indicate that an appropriate balance among cellulose fibers, silica, and boric acid is beneficial for obtaining a more continuous microstructure and a structurally stable waste-derived cementitious composite. It should be noted, however, that the SEM observations presented in this study are qualitative in nature and based on the examined fracture surfaces. Therefore, descriptions such as “relatively compact” and “relatively loose” represent comparative morphological observations rather than quantitative measures of pore structure. A comprehensive evaluation of pore size distribution and total porosity would require complementary characterization techniques, such as mercury intrusion porosimetry (MIP), which were beyond the scope of the present study.

The EDX results summarized in Table 6 show that the composites are mainly composed of C, O, Ca, and Si, which is consistent with a cellulose-modified cementitious system. The persistent dominance of Ca and O across all samples indicates that the mineral backbone of the cement matrix remains preserved after additive incorporation. The presence of Si in all modified formulations confirms the incorporation of silicate-containing phases originating from both cement hydration products and the added silica. Several modified samples exhibit relatively higher carbon contents than the reference cement, supporting the successful incorporation of cellulose-derived organic domains into the composite. Although the Si signal does not vary strictly in proportion to the nominal silica dosage, this behavior is consistent with the heterogeneous local distribution commonly encountered in cement-based composites. Similarly, sulfur signals are attributed to sulfate-containing cement constituents rather than additive-specific contributions. Boron was not distinctly detected, which is expected considering the known limitations of EDX for detecting low-concentration light elements. Overall, the EDX results support the preservation of the inorganic cement framework together with the successful incorporation of cellulose fibers and silica into the modified composite structure, while direct identification of the individual contribution of each component is beyond the capability of EDX analysis.

**Table 6:** EDX elemental composition (wt%) of the reference cement (sample 0) and recycled cellulose fiber reinforced cementitious composites

Sample	C (%)	O (%)	Si (%)	S (%)	Ca (%)	Al (%)	Fe (%)
0	22.22	37.09	4.46	3.05	30.06	1.04	1.54
1	34.14	37.59	3.55	3.34	19.58	0.82	0.75
2	25.03	36.93	4.56	4.17	26.13	1.12	1.49
3	22.61	38.2	3.99	3.89	28.95	1.01	1
4	6.59	44.97	3	3.05	42.35	0.01	0.05
5	18.94	38.27	4.45	4.1	31.07	1.13	1.7
6	17.13	42.01	3.69	1.53	35.1	0.09	0.45
7	18.89	44.62	2.81	4.06	29.62	0	0
8	19.74	43.84	3.42	1.94	30.89	0.14	0.03
9	28.26	39.89	2.45	3.96	23.38	0.81	1.13

### 3.4. Thermal analysis

**Fig. 8:**
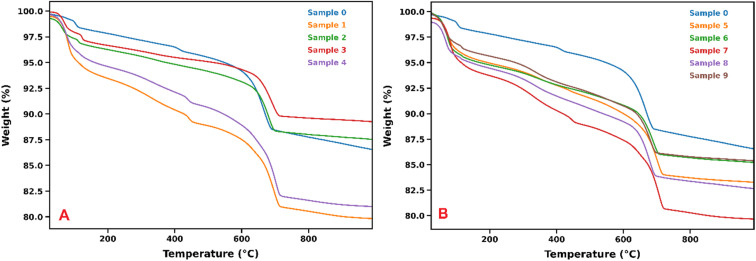
TG curves of the reference cement (sample 0) and recycled cellulose fiber reinforced cementitious composites with different boric acid and silica levels: (A) samples 1–4 and (B) samples 5–9.

The thermal behavior of the reference cement (sample 0) and the modified fiber–reinforced composites (samples 1–9) was evaluated by TG and DSC analyses, and the resulting profiles reveal a composition-dependent response influenced primarily by cellulose incorporation and additive distribution. As shown in the thermogravimetric curves in Fig. 8, all samples exhibit an initial limited mass loss of approximately 1–2% below 150 °C, corresponding to the removal of physically adsorbed moisture. A more pronounced divergence appears beyond ∼200 °C, where the modified composites display an earlier onset of mass loss relative to the reference cement. This behavior is primarily associated with the thermal decomposition of cellulose together with the dehydration of cement hydration products. Samples containing higher fiber contents exhibit total mass losses in the range of ∼17–20%, whereas the reference cement shows a lower overall loss of ∼12–14%. Although all modified formulations follow a similar decomposition pathway, differences of approximately ∼1–3% are observed among samples with comparable fiber contents. These variations are consistent with the combined influence of boric acid and silica on the thermal response of the composite system, although their individual contributions cannot be distinguished solely from the present TG results. In the higher temperature region (∼600–750 °C), corresponding to the decomposition of cementitious mineral phases, the modified composites retain final residues in the range of ∼80–85%, compared with ∼86–88% for the reference matrix. Overall, the TG results indicate that cellulose incorporation increases the overall thermal sensitivity of the composites, whereas the presence of mineral additives is associated with partial stabilization of the residual mass at elevated temperatures.^49,50^

**Fig. 9:**
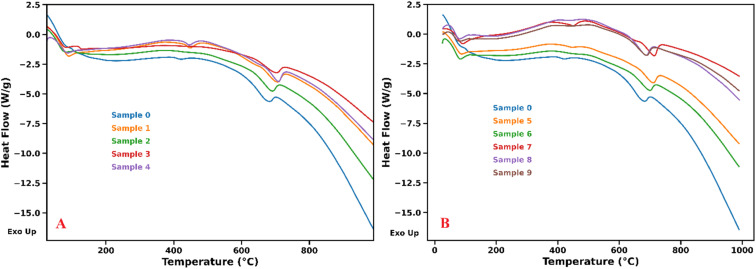
Comparative DSC curves of the reference cement (sample 0) and recycled cellulose fiber reinforced cementitious composites with different boric acid and silica levels: (A) samples 1–4 and (B) samples 5–9.

The DSC curves shown in Fig. 9 further support these thermal characteristics. All formulations display a weak low-temperature endothermic event below 150 °C, which is attributed to moisture removal. Beyond ∼200 °C, the modified composites exhibit more pronounced thermal deviations than the reference system, particularly in fiber-rich formulations, reflecting the increased contribution of cellulose-containing domains during heating. In the high-temperature region, the composite samples exhibit deeper endothermic minima than sample 0, indicating greater energy absorption during thermal decomposition. However, no distinct peak shift is observed among the modified formulations. Instead, only moderate variations in the magnitude and distribution of the heat-flow profiles are observed, which may reflect the combined influence of boric acid and silica on the thermal behavior of the composite system. Accordingly, the DSC results support that the overall thermal response is governed by the combined effects of cellulose fibers and mineral additives, while the individual contribution of each component cannot be unequivocally resolved from the present thermal analyses alone.^34,51^

The combined results of the structural characterization, and thermal analyses indicate that the incorporation of boric acid does not produce detectable changes in the principal crystalline framework of the cementitious matrix, while modest microstructural and thermal variations are observed among the modified formulations. These observations are consistent with the behavior reported for boric acid-containing cementitious systems in previous studies. Nevertheless, within the scope of the characterization techniques employed in the present work, they should be interpreted as indirect evidence rather than direct verification of the specific role of boric acid during cement hydration. Accordingly, the discussion presented herein focuses on the overall behavior of the composite system instead of attributing the observed responses to an independently verified mechanistic effect of boric acid. Accordingly, the influence of boric acid discussed in the present study should be interpreted in terms of its contribution to the overall performance of the composite system rather than as a direct assessment of cement hydration kinetics.

### 3.5. XRD analysis

**Fig. 10:**
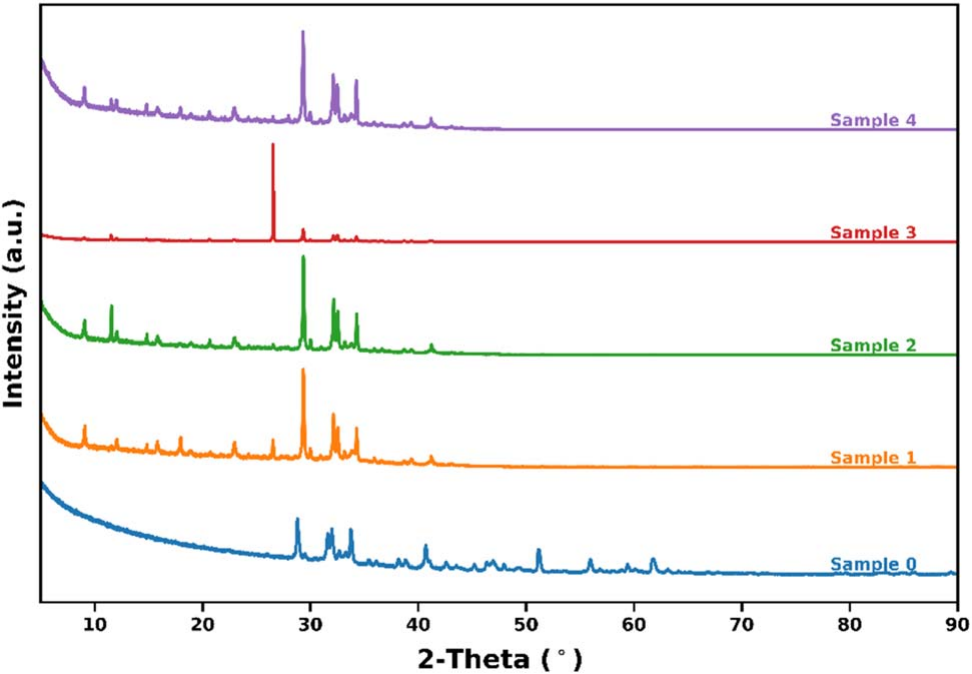
XRD patterns of the reference cement (sample 0) and recycled cellulose fiber reinforced cementitious composites: samples 1–4.

**Fig. 11:**
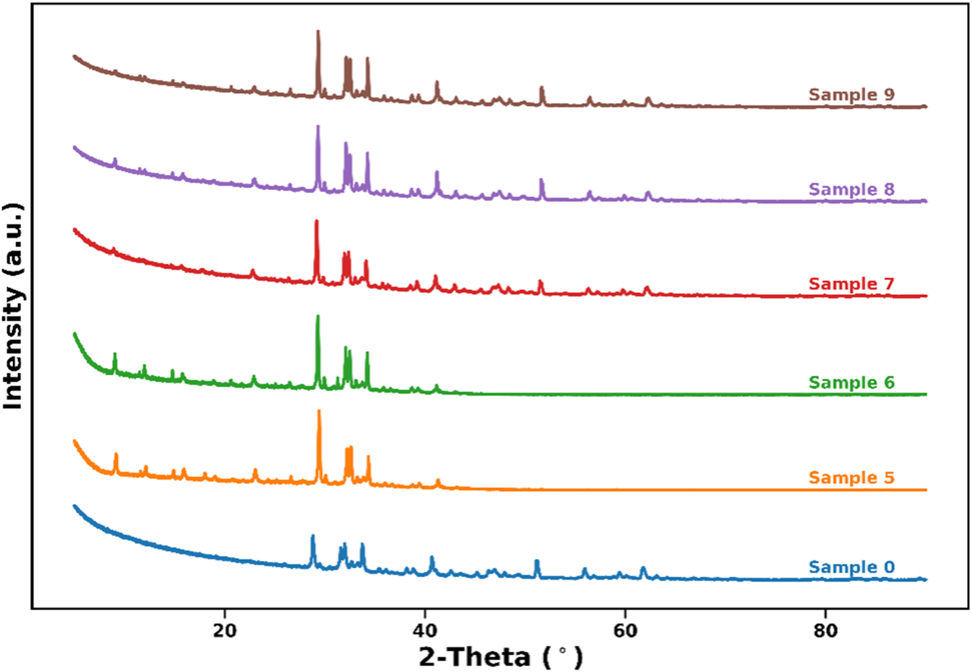
XRD patterns of the reference cement (sample 0) and recycled cellulose fiber reinforced cementitious composites: samples 5–9.

The XRD patterns of the reference cement (sample 0) and the recycled cellulose fiber–reinforced composite systems (samples 1–9), presented in Fig. 10 and 11, indicate that the principal crystalline phases of the cementitious matrix remain largely preserved after the incorporation of recycled cellulose fibers, boric acid, and silica. The dominant diffraction reflections observed around 2*θ* ≈ 29–30°, 32–34°, and 50–60° are characteristic of hydrated cement phases, mainly calcium silicate hydrate-related mineral domains and calcium hydroxide, confirming that no major changes in the crystalline phase assemblage occur after the incorporation of the additives.^52^

As shown in Fig. 10, the reference cement (sample 0) exhibits a relatively moderate main reflection at approximately 2*θ* ≈ 29.4°, whereas several modified samples display comparatively sharper and more intense diffraction profiles in this region. This observation indicates variations in the relative diffraction intensity among the modified formulations; however, the present XRD analysis does not permit direct quantification of changes in crystallinity, hydration degree, or mineral phase content. Samples containing higher silica levels exhibit relatively more pronounced diffraction profiles in the main reflection region, which is consistent with the presence of silica-containing mineral phases within the composite.^53^ Likewise, formulations containing higher cellulose contents exhibit slight peak broadening and local reductions in diffraction intensity, which may be associated with the increased amorphous contribution of cellulose-rich domains.^51^ The diffraction profiles of samples containing different boric acid contents remain generally comparable, with only minor variations in peak sharpness and baseline characteristics. These observations suggest that the incorporation of recycled cellulose fibers, silica, and boric acid does not substantially alter the principal crystalline phases of the cementitious matrix within the detection capability of the present XRD analysis.^54^

As shown in Fig. 11, all modified formulations maintain diffraction profiles similar to that of the reference cement without generating additional crystalline reflections. Therefore, the XRD results indicate that the primary crystalline framework of the cementitious matrix is preserved following additive incorporation. Although the observed differences in diffraction intensity are consistent with the microstructural variations discussed in the SEM analysis, the present XRD results alone do not provide direct evidence for the individual effects of silica on matrix densification or boric acid on hydration regulation. Accordingly, these aspects are interpreted in conjunction with the complementary SEM, thermal analysis, and relevant literature rather than being inferred solely from the XRD data.

### 3.6. Multi-response optimization of sustainable cementitious composite performance

A comprehensive evaluation of the developed recycled cellulose fiber reinforced cementitious composites was carried out in terms of compressive strength, heat capacity, thermal conductivity, and water absorption in order to identify the most balanced multifunctional formulation. For this purpose, a Taguchi-based experimental design combined with TOPSIS multi-response optimization was employed to evaluate the simultaneous effects of cellulose fiber, boric acid, and silica contents on composite performance.

The optimization procedure was based on nine experimental formulations, each tested in duplicate. The detailed duplicate results obtained from the repeated measurements are provided in Table S1 (SI), while the arithmetic mean values used for the subsequent optimization analysis are summarized in Table 7. According to these averaged data, compressive strength varied from 20.5 to 38.73 MPa, heat capacity from 1.745 to 2.412 MJ m^−3^ K^−1^, thermal conductivity from 0.613 to 0.718 W m^−1^ K^−1^, and water absorption from 16.41 to 18.15%. These variations indicate that the simultaneous incorporation of recycled cellulose fibers, boric acid, and silica produces measurable changes in both mechanical and thermophysical properties of the cementitious composites.

**Table 7:** Arithmetic mean values of compressive strength, heat capacity, thermal conductivity, and water absorption used for the multi-response optimization of recycled cellulose fiber reinforced cementitious composites

Exp. no.	Results			
	*R*_1_ (MPa)	*R*_2_ (MJ m^−3^ K^−1^)	*R*_3_ (W m^−1^ K^−1^)	*R*_4_ (%) WA
	Series average	Series average	Series average	Series average
0	23.52	1.740	0.723	18.2
1	20.5	1.745	0.718	17.80
2	29.4	1.798	0.706	18.15
3	38.73	2.412	0.628	16.41
4	28.02	1.975	0.680	17.12
5	35.9	2.385	0.613	16.58
6	24.2	1.821	0.701	17.5
7	34.5	2.144	0.665	16.72
8	30.5	1.810	0.687	17.25
9	32.3	2.112	0.674	16.89

**Fig. 12:**
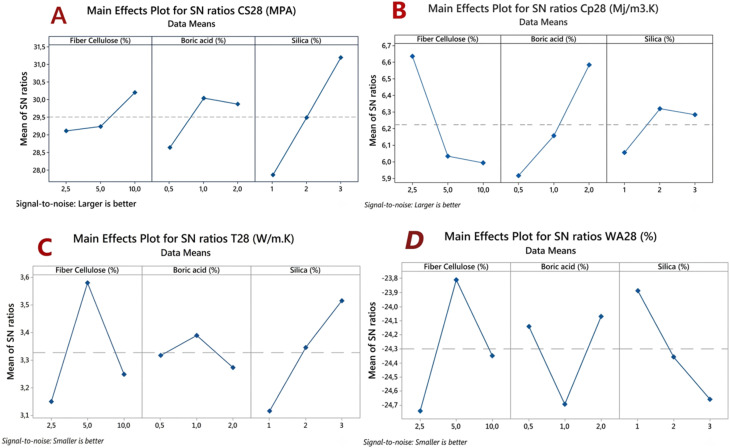
Main effect plots of S/N ratios obtained from the Taguchi analysis for the investigated performance criteria of recycled cellulose fiber reinforced cementitious composites: (A) compressive strength, (B) heat capacity, (C) thermal conductivity, and (D) water absorption.

To integrate these multiple quality criteria into a unified response parameter, the signal-to-noise (S/N) ratios obtained from the Taguchi method were processed through the TOPSIS methodology, and the resulting normalized decision matrix together with the corresponding closeness coefficients are presented in Table 8. The individual parameter trends derived from the S/N main effect plots are illustrated in Fig. 12. As shown in these plots, silica exhibits the most pronounced positive contribution to compressive strength, whereas heat capacity, thermal conductivity, and water absorption display comparatively narrower but composition-sensitive fluctuations. In particular, the progressive increase in compressive strength with increasing silica level is consistent with the relatively denser mineral morphology previously observed in the SEM and XRD analyses.

**Table 8:** TOPSIS normalized decision matrix and corresponding closeness coefficients derived from the S/N ratios of the L9(3^3^) experimental design

Decision matrix (S/N ratios)					Weight normalized decision matrix							
Response	*R*_1_	*R*_2_	*R*_3_	*R*_4_		*v*_*i*1_	*v*_*i*2_	*v*_*i*3_	*v*_*i*4_	S*	*S* ^−^	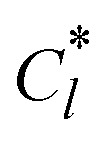
Weights	0.25	0.25	0.25	0.25								
Exp.-1	26.235	4.835	2.877	−25.00		0.0739	0.065	0.069	−0.084	0.0529	0.0005	0.0106
Exp.-2	29.366	5.095	3.023	−25.17		0.0827	0.06	0.072	−0.085	0.0461	0.0101	0.1801
Exp.-3	31.760	7.647	4.040	−24.19		0.0895	0.103	0.097	−0.081	0.0050	0.0499	0.9079
Exp.-4	28.949	5.911	3.349	−24.67		0.0815	0.080	0.080	−0.0833	0.0330	0.0201	0.3781
Exp.-5	31.101	7.549	4.250	−24.39		0.0876	0.102	0.102	−0.082	0.0023	0.0514	0.9558
Exp.-6	27.676	5.206	3.085	−24.86		0.0779	0.070	0.074	−0.083	0.0449	0.0082	0.1549
Exp.-7	30.756	6.624	3.543	−24.46		0.0866	0.089	0.085	−0.082	0.0221	0.0318	0.5894
Exp.-8	29.686	5.153	3.260	−24.73		0.0836	0.069	0.078	−0.083	0.0418	0.0141	0.2529
Exp.-9	30.184	6.493	3.426	−24.55		0.0850	0.088	0.082	−0.082	0.1694	0.0284	0.1438
	88.708	18.42	10.36	74.02	A**	0.0895	0.1037	0.1024	−0.0817			
					*A* ^−^	0.0739	0.0656	0.0693	−0.0850			

**Fig. 13:**
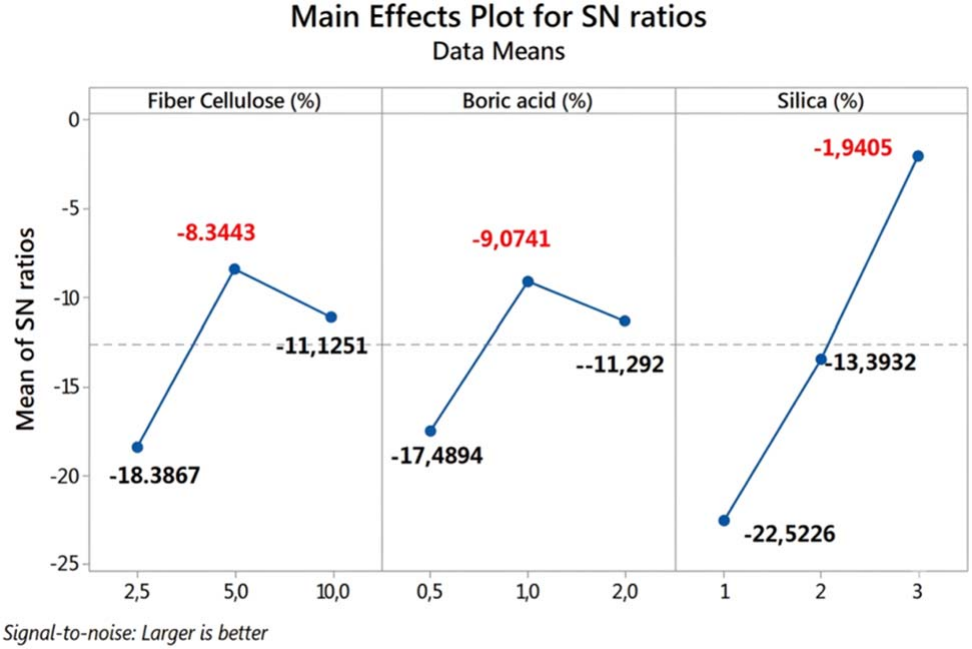
Main effect plots of weighted normalized S/N ratios obtained from TOPSIS-based multi-response optimization, showing the optimum parameter levels for recycled cellulose fiber reinforced cementitious composites.

Based on the weighted normalized S/N ratios presented in Fig. 13, the Taguchi–TOPSIS analysis identified the A2B2C3 formulation as the optimum combination among the experimentally evaluated formulations in the L9 orthogonal array. This formulation corresponds to 5 g recycled cellulose fiber, 1 g boric acid, and 3 g silica. This formulation yielded the highest overall closeness coefficient and therefore the most balanced multifunctional response among all tested systems. A more detailed statistical evaluation of the factor effects is given in Table 9, where silica was identified as the only parameter exerting a statistically significant influence on compressive strength (*T* = 6.30; *p* < 0.001), while cellulose fiber and boric acid acted as secondary formulation modifiers. In contrast, the remaining thermal and physical performance criteria did not show statistically significant factor sensitivity within the investigated parameter range.

**Table 9:** Statistical evaluation of factor effects based on *T* and *p* values for the investigated performance criteria of recycled cellulose fiber reinforced cementitious composites

Criteria parameters	CS28		CP28		T28		WA28	
	*T*	*P*	*T*	*P*	*T*	*P*	*T*	*P*
Fiber cellulose (%)	1.77	0.098	−1.23	0.240	−0.07	0.947	−0.48	0.638
Boric acid (%)	1.96	0.070	0.99	0.341	0.23	0.821	−0.45	0.662
Silica (%)	6.30	0.000	0.49	0.631	−1.75	0.103	1.55	0.143

The performance enhancement ratios of the optimum formulation (A2B2C3), identified through the Taguchi–TOPSIS analysis and experimentally evaluated within the L9 orthogonal array, are summarized in Table 10. Compared with the reference cement matrix, the A2B2C3 composite exhibited a 52.63% increase in compressive strength and a 37.06% increase in heat capacity, together with a 15.21% reduction in thermal conductivity and an 8.90% reduction in water absorption. These findings demonstrate that a carefully balanced additive design enables the sustainable conversion of recycled cellulose waste into a mechanically strengthened and thermally improved cementitious composite with multifunctional performance advantages.

**Table 10:** Performance improvement ratios of the optimized recycled cellulose fiber reinforced cementitious composite relative to the reference cement matrix

Response	Reference cement matrix (sample 0)	Optimized A2B2C3 composite (sample 5)	Improvement ratio (%)
Compressive strength, CS28 (MPa)	23.52	35.90	+52.63
Specific heat capacity, CP28 (MJ m^−3^ K^−1^)	1.740	2.385	+37.06
Thermal conductivity, T28 (W m^−1^ K^−1^)	0.723	0.613	−15.21
Water absorption, WA28 (%)	18.20	16.58	−8.90

Beyond the thermal characteristics discussed above, the incorporation of recycled cellulose fibers should also be considered from the perspective of the overall performance of the developed composites. Although the TG results indicate a moderate reduction in thermal stability, this behavior should be interpreted in the context of the multifunctional role of cellulose within the cementitious matrix. In the present study, recycled cellulose fibers primarily act as a sustainable reinforcing phase, contributing to improved mechanical performance, favorable thermal insulation characteristics, and the valorization of paper industry waste. These functional benefits provide an important advantage for the development of eco-efficient cementitious materials despite the increased thermal sensitivity associated with the organic nature of cellulose. Furthermore, the cellulose fibers are embedded within a predominantly inorganic cementitious matrix, where the surrounding hydration products may limit their direct exposure to external environmental factors. Consequently, although cellulose is inherently more susceptible to thermal, biological, and chemical degradation than the cement matrix itself, its incorporation at the investigated levels represents a reasonable balance between enhanced multifunctional performance and the moderate reduction in thermal stability. Nevertheless, comprehensive long-term durability studies under aggressive environmental conditions are still required to fully evaluate the biological and chemical stability of cellulose-containing cementitious composites. Such investigations will provide a more comprehensive understanding of the long-term performance of these sustainable cement-based materials and constitute an important direction for future research.

## 4. Conclusion

This study demonstrates the sustainable conversion of recycled cellulose fibers into boric acid/silica modified cementitious composites through a Taguchi–TOPSIS multi-response optimization strategy. Thermal analyses revealed that cellulose incorporation increases the overall thermal responsiveness of the cement matrix, while boric acid and silica contribute to a more composition-sensitive residual behavior. SEM-EDX and XRD results further showed that the principal mineral framework of the cementitious system remains preserved after additive incorporation, with silica-containing formulations exhibiting a relatively denser structural distribution. Among the investigated parameters, silica was identified as the most influential factor governing the multifunctional performance of the composites. The A2B2C3 formulation, identified as the optimum experimentally evaluated composition through the Taguchi–TOPSIS multi-response optimization, provided a 52.63% increase in compressive strength and a 37.06% increase in heat capacity, together with reduced thermal conductivity and water absorption relative to the reference cement matrix. Overall, the findings establish an effective waste-to-value material design route for producing mechanically strengthened and thermally improved sustainable cementitious composites. Future studies may further extend this approach through long-term durability evaluation, life-cycle assessment, and scale-up processing investigations.

## Conflicts of interest

There are no conflicts to declare.

## Supplementary Material

RA-016-D6RA04542A-s001

## Data Availability

The data supporting the findings of this study are available from the corresponding author upon reasonable request. Supplementary information (SI): the detailed mathematical formulation and step-by-step calculations of the TOPSIS multi-response optimization procedure. See DOI: https://doi.org/10.1039/d6ra04542a.
