# Supplementary material for: Sustainable valorization of recycled cellulose fibers into boric acid/silica modified cementitious composites via multi-response optimization
Source: RSC Adv. 2026 Jul 9;16(36):38756–72. doi: 10.1039/d6ra04542a (PMC13346872; doi:10.1039/d6ra04542a)
Supplement: Supplementary file 1 [file RA-016-D6RA04542A-s001.pdf]

Supplementary Information (SI) for RSC Advances.
This journal is © The Royal Society of Chemistry 2026

SUPPORTING INFORMATION

Sustainable Valorization of Recycled Cellulose Fibers into Boric Acid/Silica Modified Cementitious Composites via Multi-Response Optimization

Fehmi SALTAN[1*], Mücahit UĞUR[2], Ayça GÜNAY[1], Medine Nur KARAKURT[1], Fatih TURAN[3], Mehmet ÖZDOĞAN[3], Şahin GÜLERYÜZ[3]

[1]Faculty of Science, Department of Chemistry, Cankiri Karatekin University, Çankırı, Turkey

[2]Department of Chemistry Engineering, Faculty of Engineering, Cankiri Karatekin University, Çankırı, Turkey

[3]Department of Mining Engineering, Faculty of Engineering, Dokuz Eylul University, İzmir, Turkiye

fehmisaltan@karatekin.edu.tr

https://orcid.org/0000-0002-9347-6416

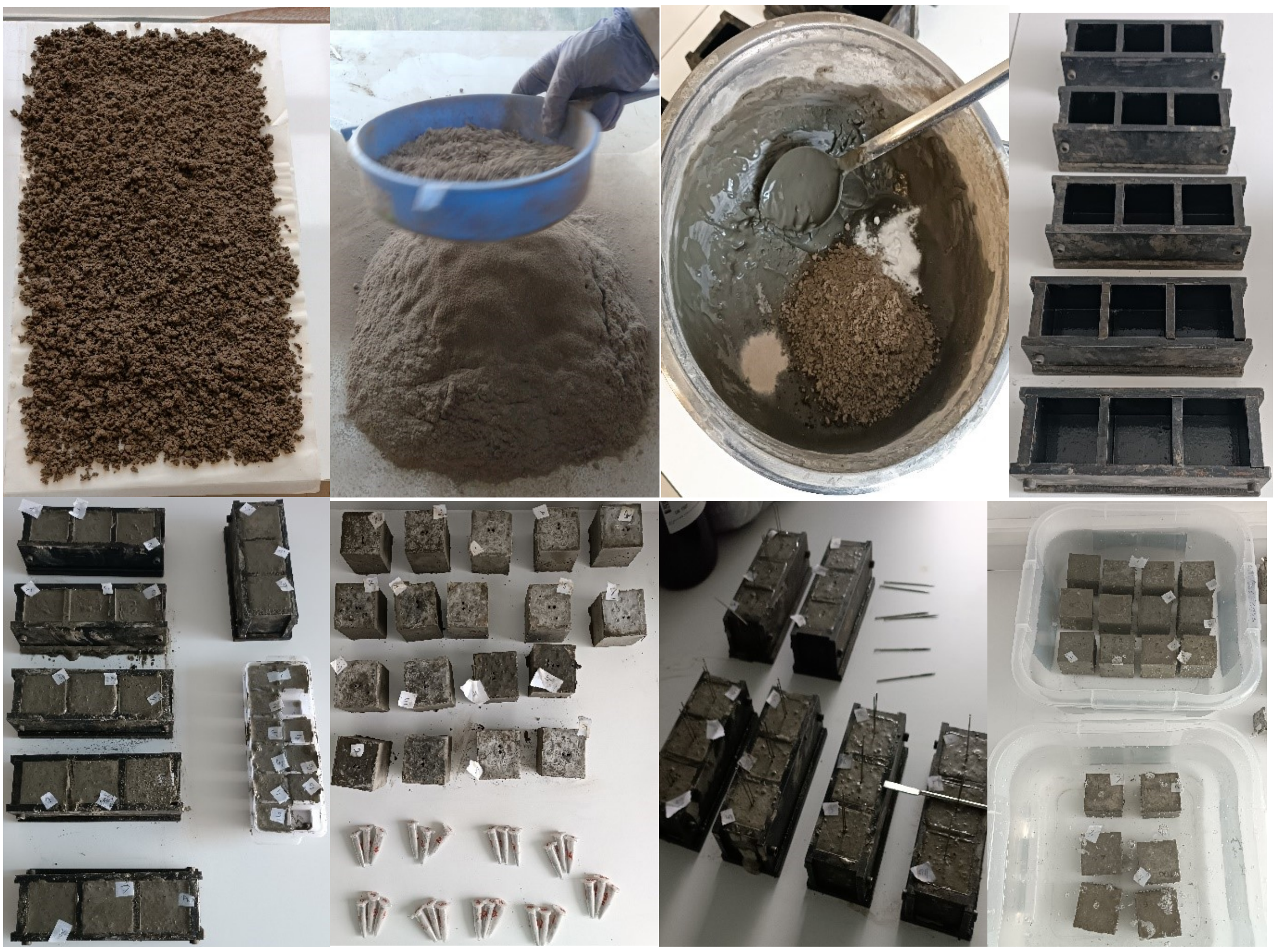

SF1. Production of Fiber-Based Cement Composites and Experimental Procedure Stages

Table S1. Duplicate experimental results of compressive strength, heat capacity, thermal conductivity, and water absorption for recycled cellulose fiber reinforced cementitious composites.

Sample No	**R1** (MPa)		**R2** (MJ/m³.K)		**R3** (W/m.K)		**R4** (%) WA	
	**1st series**	**2nd series**	**1st series**	**2nd series**	**1st series**	**2nd series**	**1st series**	**2nd series**
0	22,2	24,8	2,144	1,742	0,718	0,765	18,15	14,8
1	19,5	21,5	2,385	1,798	0,706	0,73	16,21	15,25
2	27	31,8	1,975	2,412	0,699	0,705	17,8	19,43
3	36	41,5	2,144	2,385	0,624	0,71	16,21	18,76
4	26	30,04	1,81	2,144	0,625	0,665	16,58	13,98
5	34	37,8	2,412	1,745	0,612	0,681	16,58	16,32
6	23	25,4	1,975	2,144	0,69	0,7	16,72	12,5
7	33	36	1,81	2,112	0,682	0,69	16,72	17,9
8	29	32	2,112	1,81	0,675	0,69	16,89	16,04
9	30,8	33,8	2,1	2,1	0,69	0,7	16,89	14,5

The steps in the TOPSIS methodology enable multiple criteria into a single performance response.

Step 1: The decision matrix is constructed as follows; The decision points and criteria in the experimental design represent the rows andcolumns of the matrix. In the matrix in Equation 2.3, m represents the number of alternatives, and n represents the evaluation factors (criteria).

$$A_{ij} = \begin{bmatrix} a_{11} & a_{12} & \cdots & a_{1n} \\ a_{21} & a_{22} & \cdots & a_{2n} \\ . & & & . \\ . & & & . \\ . & & & . \\ a_{m1} & a_{m2} & \cdots & a_{mn} \end{bmatrix} \qquad \text{Eq.1}$$

Step 2: Creation the standard decision matrix, Equation 2;

$$r_{ij} = \frac{a_{ij}}{\sqrt{\sum_{k=1}^{m} a_{kj}^2}} \qquad i= 1, \ldots\ldots m \text{ ve } j= 1, \ldots\ldots\ldots n \qquad \text{Eq.2}$$

Step 3: Creating the Weighted Standard Decision Matrix, Equation 3;

$$V_{ij} = \begin{bmatrix} w_1 r_{11} & w_2 r_{12} & \cdots & w_n r_{1n} \\ w_1 r_{21} & w_2 r_{22} & \cdots & w_n r_{2n} \\ . & & & . \\ . & & & . \\ . & & & . \\ w_1 r_{m1} & w_2 r_{m2} & \cdots & w_n r_{mn} \end{bmatrix} \quad \sum_{i=1}^{n} w_i = 1 \qquad \text{Eq.3}$$

Step 4: Generating Ideal (A*) and Negative Ideal (A-) Solutions; The positive ideal solution is based on the maximum S/N ratio, while the negative ideal solution is based on the minimum S/N ratio. The calculated S_i^* and S_i^- values are equal to the number of outputs in the experimental design.

$$A^* = \left\{ (\max_i v_{ij} \,|\, j \in J), (\min_i v_{ij} \,|\, j \in J') \right\} \qquad \text{Eq.4}$$

$$A^- = \left\{ (\min_i v_{ij} \,|\, j \in J), (\max_i v_{ij} \,|\, j \in J') \right\} \qquad \text{Eq.5}$$

Step 5: Calculation of Discrimination Measures; in the TOPSIS method, in the decision matrix (S_i^*) represents the distance to the positive ideal solution and (S_i^-) represents the distance to the negative ideal solution calculated using Equations 6 and 7.

$$S_i^* = \sqrt{\sum_{j=1}^{n} (v_{ij} - v_j^*)^2} \qquad \text{Eq.6}$$

$$S_i^- = \sqrt{\sum_{j=1}^{n} (v_{ij} - v_j^-)^2} \qquad \text{Eq.7}$$

Step 6 Calculating the Relative Distance to the Ideal Solution; The relative distance of the decision points to the ideal solution is calculated using the Ci* value given in Equation 8. It is determined by utilizing the ideal and negative ideal separation measures.

$$Ci^* = \frac{S_i^-}{S_i^- + S_i^*} \qquad \text{Eq.8}$$

Here, Ci* values are those within the range $0 \le Ci^* \le 1$.
